# The Role of Nrf2 in Relieving Cerebral Ischemia-Reperfusion Injury

**DOI:** 10.2174/1570159X21666221129100308

**Published:** 2023-05-12

**Authors:** Yu Sun, Xu Yang, Lijun Xu, Mengxiao Jia, Limeng Zhang, Peng Li, Pengfei Yang

**Affiliations:** 1 Henan International Joint Laboratory of Cardiovascular Remodeling and Drug Intervention, College of Pharmacy, Xinxiang Medical University, Xinxiang Key Laboratory of Vascular Remodeling Intervention and Molecular Targeted Therapy Drug Development, Xinxiang, 453003, China;; 2 School of Nursing, Pingdingshan Polytenchnic College, Pingdingshan, 467001, China

**Keywords:** Stroke, ischemic stroke, I/R, Nrf2, oxidative stress, hormesis

## Abstract

Ischemic stroke includes two related pathological damage processes: brain injury caused by primary ischemia and secondary ischemia reperfusion (I/R) injury. I/R injury has become a worldwide health problem. Unfortunately, there is still a lack of satisfactory drugs for ameliorating cerebral I/R damage. Nrf2 is a vital endogenous antioxidant protein, which combines with Keap1 to maintain a dormant state under physiological conditions. When pathological changes such as I/R occurs, Nrf2 dissociates from Keap1 and activates the expression of downstream antioxidant proteins to exert a protective effect. Recent research have shown that the activated Nrf2 not only effectively inhibits oxidative stress, but also performs the ability to repair the function of compromised mitochondria, alleviate endoplasmic reticulum stress, eliminate inflammatory response, reduce blood-brain barrier permeability, inhibit neuronal apoptosis, enhance the neural network remolding, thereby exerting significant protective effects in alleviating the injuries caused by cell oxygen-glucose deprivation, or animal cerebral I/R. However, no definite clinical application report demonstrated the efficacy of Nrf2 activators in the treatment of cerebral I/R. Therefore, further efforts are needed to elaborate the role of Nrf2 activators in the treatment of cerebral I/R. Here, we reviewed the possible mechanisms underlying its potential pharmacological benefits in alleviating cerebral I/R injury, so as to provide a theoretical basis for studying its mechanism and developing Nrf2 activators.

## 1 INTRODUCTION

Stroke refers to the immediate deficiency of local brain blood supply caused by cerebral vascular rupture or arterial embolism, which could cause subsequent neuron impairment and neurological dysfunction or even the loss of life. It is reported that stroke is the leading cause of long-term disability and also the 5^th^ leading cause of mortality, which costs up to nearly 34 billion dollars each year in the US [1], therefore poses great threats to public health and creates heavy socioeconomic burdens.

There are 86 billion neurons and more than 250-300 billion glial cells in the brain, making brain the organ bearing the largest oxygen consumption. Together with its relatively insufficient energy reserve, the brain function depends greatly on blood supply and is extremely sensitive to ischemia [2]. Unequal blood supply in different regions is the intrinsic factor for various injury degrees for neurons after cerebral ischemia. In the core area, rapid irreversible damages occur upon the neurons because of a sudden blocked energy supply and subsequent ionic failure. However, the neurons in the ischemic penumbra mostly face mild and reversible injury due to partial blood supply originating from collateral circulation. Therefore, timely restoring blood supply is an essential therapeutic method to relieve the injury of neurons in the ischemic penumbra after ischemic stroke [3]. At present, the widely accepted interventions are tissue plasminogen activator administration and mechanical thrombectomy [4]. However, it is found that after blood perfusion, the neurons in the penumbra would face a greater challenge-the cerebral ischemia/reperfusion (I/R) injuries, which appear as ion disorder, calcium overload, mitochondrial dysfunction, oxidative stress, glutamate toxicity, neuro-inflammation, programmed cell death, and finally acute impairment of neurobehavioral function [5]. Briefly, stroke is a kind of disease, while cerebral I/R injury is an inevitable pathophysiological change secondary to the essential perfusion treatment for ischemic stroke. Cerebral I/R injury is based on the primary ischemic injury but is more complex than ischemic brain injury, and its impact on the prognosis of patients is more serious. In conclusion, cerebral I/R poses serious damage to neurons in the penumbra, however, there is still a lack of effective therapeutic drugs. Therefore, it is particularly urgent to find specific targets and develop drugs to distinctively alleviate cerebral I/R injuries according to their pathogenesis.

**Fig. (1):**
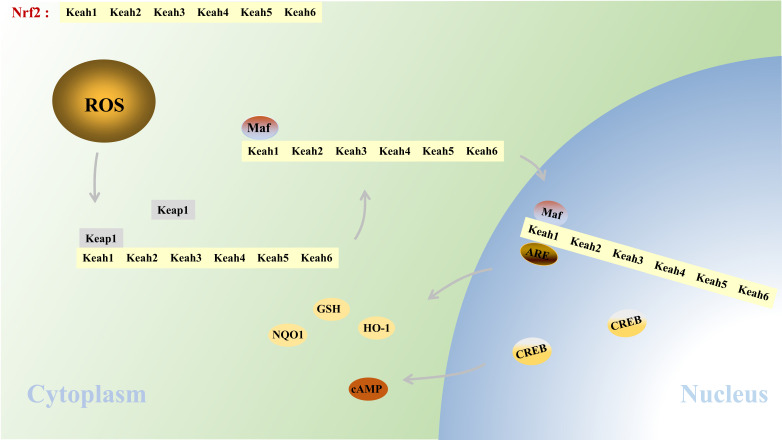
The activation of Keap1- Nrf2 pathway. **Abbreviations**: Nrf2: Nuclear factor E2-related factor 2; Keap1: Kelch-like ECH associated protein-1; ARE: antioxidant response element; HO-1: heme oxygenase-1; ROS: reactive oxygen species; NQO1: NAD(P)H: Quinone oxidase 1; GSH: glutathione.

Nuclear factor erythroid 2-related factor 2 (Nrf2), consisting of six characteristic domains: Keah1, Keah2, Keah3, Keah4, Keah5 and Keah6, is a member of CNC (Cap ‘n’ Collar) transcription factor family to modulate the transcription and translation of various endogenous antioxidant elements. Under physiological conditions, Kelch-like ECH-associated protein-1 (Keap1) combines with Keah2 and Keah6 domains of Nrf2 and degrades Nrf2 through the ubiquitination complex-dependent pathway to keep Nrf2 in a dormant state. After cerebral I/R, Keap1 is degraded and thus loses its inhibitory effects on Nrf2, making Nrf2 activated. The activated Nrf2 forms a heterodimer with Maf protein and then combines with antioxidant response element (ARE), subsequently elevating the expression of downstream antioxidant proteins like heme oxygenase-1 (HO-1), glutathione family (GSH) and NAD(P) H: Quinone oxidase 1 (NQO1), all of which could mitigate oxidative stress through scavenging free radicals (Fig. **1**). Furthermore, the activated Nrf2 also binds cyclic adenosine 5‘-monophosphate (AMP)-responsive element binding protein (CREB) through its Neh4 and Neh5 domains, enhancing the phosphorylation of CREB to regulate the survival of neurons, subsequently leading to the alleviation of neuron injury after cerebral I/R [6, 7]. The high expression of Nrf2 has been proved to probably play a protective role on various cells in the central nerve system (CNS) after ischemia: 1. The diverse expression levels of Nrf2 among various components of the nervous system are negatively correlated with the degrees of cell injury. For example, compared with astrocytes which show high expression of Nrf2, severer hypoxic-ischemic damage was observed in oligodendrocytes and pericytes, which show relatively limited expression of Nrf2 [8]. An animal experiment also confirmed that resveratrol treatment induced a protective effect after cerebral ischemia through activating Nrf2, while this protection was weakened in Nrf2^-/-^ group, along with decreased concentrations of antioxidant proteins thioredoxin (Trx) and NQO1 and enhanced reactive oxygen species (ROS) contents in their astrocytes [9]. 2. The high expression of Nrf2 might suppress the cell death process. For example, the core ischemic zone where no Nrf2 staining was found showed massive cell death, and the significantly enhanced Nrf2 expression in glial and neuronal cells might be the reason for the mild injury in ischemic penumbra cells [10]. In addition, an oxygen-glucose deprivation/reperfusion (OGD/R) experiment using primary neurons also confirmed that the activation of Nrf2 could alleviate oxidative stress and neuronal apoptosis occurred after reperfusion while repressing the transcription activity of Nrf2 would reverse its protective effects [11]. Similarly, several animal cerebral I/R experiments also revealed that activating Nrf2 could remarkably decrease the infarct volume of the brain and alleviate neurological dysfunction while knocking out Nrf2 gene would obviously hinder this protection [12-14]. Here, we review the possible mechanisms of Nrf2 in relieving injuries from the view of the pathological mechanisms and processes of cerebral I/R injuries and hope to provide theoretical basis for considering Nrf2 as a novel target in developing drugs for cerebral I/R. To systematically explore the protective effects of Nrf2 in different stages of cerebral ischemia, we review its role according to the chronological order, including ischemic preconditioning (IPC) before the occurrence of ischemia, at the stage of I/R and the long-term prognosis after I/R, in order to introduce the role of Nrf2 in cerebral I/R in detail.

## 2 Nrf2 ENHANCES THE TOLERANCE AGAINST CEREBRAL I/R IN ISCHEMIC PRECONDITIONING

IPC refers to a process that the sub-lethal degree ischemia and hypoxia are given to tissues before stroke in order to enhance the tolerance of the brain to cope with the following ischemic injuries [15]. With the deepening of understanding, it is gradually recognized that IPC is one special kind of hormesis [16]. Hormesis is characterized by a low-dose/stress (dose/stress below the threshold) stimulation and a high-dose/stress inhibition, which cannot be predicted by traditional dose/stress-response models in low-dose/stress [16]. Furthermore, the application of either subtoxic hormetic stress or subtoxic hormetic preconditioning may induce hormesis and alleviate the injuries caused by subsequent toxic doses or lethal stress [17, 18]. Hormesis is common and highly generalizable, being independent of the biological model, endpoints measured, and physical agent studied. Therefore, it is recommended that IPC is hormesis [19, 20].

How IPC protects I/R injuries? IPC was believed to modulate endogenous cellular defense activity and represent an innovative approach to alleviate tissue damage [16]. ROS is believed to be involved in modulating cellular defense activity in IPC [21]. Excessive ROS results in cellular injuries, while lower amounts of oxidative stress may induce an adaptive stress response, followed by enhanced survival [16]. Briefly, modest ROS generation induced by IPC rapidly consumes the antioxidants reserves in the brain and then regulates a series of genes related to cell stress response, which are called vitagenes. Vitagenes encode proteins related to alleviating oxidative injuries [22, 23]. These proteins include Nrf2, sirtuin [16] and heat shock proteins [22]. Of which, Nrf2 might be an important one, as Nrf2 is considered the general and dominant underlying mechanistic basis of hormesis [18] and IPC [21].

IPC is proved to enhance Nrf2 expression, therefore inhibit apoptosis, reduce brain tissue damage, diminish cerebral infarct volume, alleviate sensorimotor and cognitive deficits and protect animals from vascular dementia [15, 21, 24]. However, this phenomenon was inhibited in Nrf2 KO mice, or stimulated by a blood-brain barrier (BBB) permeable Nrf2 activator, which implicated that Nrf2 may play a vital role in the protective effect of IPC in I/R [15, 21].

### 2.1 Nrf2 Increases the Plasticity of Brain Structure in IPC

The brain is composed of white matter and grey matter, and the oligodendrocytes in white matter play a critical role in stabilizing the brain structure. Due to the weak ability of oligodendrocytes to resist oxidative stress resulting from limited expression of Nrf2, the brain structure always faces high risk after I/R. Neural stem cells (NSCs), which possess the potential to proliferate and differentiate into neurons or glial cells, exist in the subventricular zone with a dormant state under physiological conditions. Animal experiments showed that IPC could enhance the expression of Nrf2, and promote NSCs to proliferate and differentiate into oligodendrocytes in the striatum and corpus callosum, thus maintaining the stability of brain structure after cerebral I/R [15, 25].

### 2.2 Nrf2 Activation in Astrocytes Alleviates Cerebral I/R Injuries in IPC

Most studies in this field proved that Nrf2 activation in astrocytes might be the primary role in IPC protection. i) Activating Nrf2 in astrocytes protects astrocytes to exert their physiological functions. Some researchers found that the Nrf2 activated by IPC was highly expressed in astrocytes, enhanced the steadiness of mitochondrial respiratory chain and oxidative phosphorylation, protected astrocytes and rehabilitated the activity of astrocytes after OGD/R [26], while the enhancement of astrocytes function promotes the integrity of BBB [27]. These results were further proved by a study showed that IPC could weaken the inhibition of glycogen synthase kinase 3β (GSK3β) and Keap1 on Nrf2, thus enhancing the expression of Nrf2 and up-regulating the expression of cadherin, repressing the damage to BBB and eventually reducing the infarct volume of the brain [28]. ii) Activating Nrf2 in astrocytes also strengthens the supportive effect of astrocytes on neurons (including reference [26]). Bell *et al*. proved that IPC induced oxidative stress, activated Nrf2 in astrocytes to up-regulate antioxidant genes and exerted a protective effect in mixed cultures of neurons and astrocytes, however, this protection was significantly diminished in pure neuron cultures, demonstrating the locus of Nrf2 activation within the mixed cultures are centred on the astrocytes. Besides, the protective effect in IPC was observed in WT mixed cultures, however significantly diminished in the absence of Nrf2, which proved that Nrf2 plays a critical role in IPC [29, 30]. We need to mention that most of the studies showed that IPC enhanced Nrf2 expression, induced mild oxidative stress, promoted the formation of electron transport chain supercomplexes to down-regulate ROS production, activated ARE to up-regulate subsequent antioxidases such as HO-1 or sulfiredoxin 1(Srxn1), eventually alleviated I/R injuries [21, 24, 26, 29-31] (the role of Nrf2 in these protective effects will be elaborated in depth in the following part).

## 3 Nrf2 MODULATES VARIOUS PATHOLOGICAL PROCESSES IN CEREBRAL I/R

### 3.1 Nrf2 Restores Mitochondrial Function

Compared with other tissues and organs in the human body, the mitochondria are more abundant in the brain. About 90% of the energy needed for neuronal activities is generated by mitochondria, so the stability of mitochondrial function shows a close correlation with the physiological function of the neurons [32]. Mitochondria are also the regulatory hubs for a cascade of cellular functions and signaling pathways, such as the citric acid cycle, fatty acid oxidation, electron transport, calcium homeostasis, inflammation, oxidative phosphorylation, neurotransmitters storage and synaptic vesicle recycling [33]. Mitochondria are composed of bilayer membranes including the outer membrane, inner membrane and the mitochondrial matrix. Voltage-dependent anionic channels (VDACs) exist on the outer membrane and only permit the transmission of small molecules across the outer membrane. The inner membrane contains the key enzymes regulating the citric acid cycle and substrates launching oxidative phosphorylation [34].

After ischemia, mitochondrial depolarization which is induced by ion disorders leads to the accumulation of PINK1 (PTEN-induced putative kinase1) and reduction of adenosine triphosphate (ATP) content [35]. Insufficient ATP induces the generation of O_2_·^-^ that is derived from O_2_ partial reduction, increases the contents of ROS, and aggravates oxidative stress in the brain [36]. Excessive ROS directly induces mitochondrial permeability transition pore (mPTP) opening, which not only causes mitochondrial swelling and dysfunctions but also leads to the release of cytochrome c (Cyt C) and apoptosis-inducing factor (AIF) to induce or boost apoptotic cascade in neurons [37, 38]. In a cell experiment using primary neurons, it was found that the recovery of mitochondrial function was helpful for alleviating the apoptotic cascade of neurons after cerebral ischemia. In an animal study using the MCAO model, it was verified that the cerebral infarct volume in mice who were knocked out of mitochondrial phosphoglycerate mutase 5 (PGAM5) was significantly enlarged when compared with wild-type mice [39]. These results clarified that stabilizing the function of mitochondria could perform a positive effect on reducing cerebral I/R injury and recovering neurological function.

Nrf2 stabilizes the mitochondrial function of neurons by promoting mitophagy and modulating mitochondrial dynamics, subsequently playing a protective role after cerebral I/R.

#### 3.1.1 Promoting Mitophagy

Autophagy refers to the process where unneeded cytoplasmic components (including long-lived proteins and organelles, mitochondria, peroxisomes, Golgi or endoplasm) are digested by lysosomal enzymes. It always seems to be a double-edged sword. A basal autophagy is widely believed to be a beneficial approach to protect cellular homeostasis through cleaning those long-lived or misfolded proteins and damaged organelles, while excessive autophagy causes cell death [40]. Mitophagy, also known as mitochondrial autophagy, is one special kind of autophagy that could selectively eliminate injured mitochondria. Although the role of mitophagy in cerebral I/R is still in debate, it is well believed that modest mitophagy, which means the clearance of damaged mitochondria, is beneficial for neuronal survival during cerebral I/R [41].

Nrf2 can induce or facilitate mitophagy after cerebral I/R in the following ways:

##### 3.1.1.1 Promoting Mitophagy by PINK1 Pathway

PINK1 is a serine/threonine protein kinase, which attracts Parkin to the outer membrane of depolarized mitochondria and facilitates the occurrence of autophagy [42]. Nrf2 positively regulates PINK1 expression, which is critical in mediating cell survival [43]. Therefore, several studies reported that the treatment of cerebral I/R using chemicals could increase Nrf2 expression up-regulated PINK1, Parkin, Beclin1 and LC3II/I, and these results indicated that Nrf2 may ameliorate cell injury by promoting PINK1 and subsequent mitophagy in cerebral I/R [44] and subarachnoid hemorrhage [45].

##### 3.1.1.2 Enhancing p62-Mediated Mitophagy

p62 acts as an autophagy regulator and initiates mitophagy in a PINK1 independent manner. There is positive feedback between p62 and Nrf2 which facilitates mitophagy. The accumulation of ROS after cerebral I/R not only induces oxidative stress, but also up-regulates the level of p62. Then, the up-regulated p62 degrades Keap1 through ubiquitination, accelerates the dissociation between Nrf2 with Keap1, and further activates Nrf2 and Nrf2-ARE system. In addition, the activation of Nrf2 reverses the inhibition of p62 occurred after cerebral I/R, and further forms a heterodimer with p62 to recruit LC3 to the outer membrane of mitochondria, eventually inducing or enhancing mitophagy [46, 47].

#### 3.1.2 Improving Mitochondrial Dynamic Dysfunction

Mitochondrial dynamics, including mitochondrial fission, fusion and biosynthesis, act as key participants to maintain mitochondrial activities. Mitochondrial dynamics reflects the ability of mitochondria to respond to internal or external stimulation and its dysfunction is closely correlated to the occurrence of serious cardiovascular diseases [48]. Mitochondrial fission and fusion are both energy-consuming processes, which means the sharp decline of energy due to ischemia could lead to dysfunction of mitochondrial dynamics after cerebral I/R [49], while Nrf2 could alleviate these dysfunctions.

##### 3.1.2.1 Inhibiting Mitochondrial Fission

Mitochondrial fission is an event that one mitochondrion is divided into at least two mitochondria [50]. Mitochondrial fission is primarily activated because more mitochondria are needed for the metabolic needs of cells. Appropriate mitochondrial fission not only produces more daughter mitochondria to promote oxidative phosphorylation, but also permits the separation of impaired mitochondria parts, therefore, mitochondrial fission, which is mainly regulated by dynamin-related protein 1 (Drp1) and mitochondrial fission protein 1 (Fis1), is necessary for the maintenance of healthy mitochondria [51]. However, excessive mitochondrial fission decreases ATP synthesis, impairs the detoxification of ROS, disturbs the extrusion of intracellular Ca^2+^, releases pro-apoptotic factors and eventually causes apoptosis under stress conditions [52]. It is reported that excessive mitochondrial fission leads to the lethal and irreversible destruction of the mitochondria [53], which is observed during both the cerebral ischemic phase and I/R stage [54]. Moreover, the inhibition of mitochondrial fission is believed to alleviate cerebral I/R injuries [51].

Although there is no direct evidence proving that Nrf2 can inhibit mitochondrial fission to alleviate cerebral I/R injury, indirect evidence was observed. One study found that the increase of Nrf2 and inhibition of mitochondrial fission (indicated by phosphorylation of Drp1(Ser637) and dephosphorylation of Drp1(Ser616)) occurred simultaneously during the treatment of cerebral I/R [44], and another PC12 cell OGD/R model also reported that 3-n-butylphthalide treatment increased Nrf2 expression and down-regulated Drp1 and Fis1 expression, therefore inhibited mitochondrial fission and improved cell activity [55]. These results hinted that Nrf2 may be related to the regulation of mitochondrial fission in cerebral I/R. The possible relationship may lie in that enhanced ROS production during cerebral I/R induces mitochondrial fission and dysfunction [56], while the scavenging capacity of Nrf2 for ROS can reduce the occurrence of mitochondrial fission.

##### 3.1.2.2 Promoting Mitochondrial Fusion

Mitochondrial fusion is a process in which two mitochondria merge into a larger mitochondrion. Mitochondrial fusion facilitates the maintenance of mitochondrial morphology, promotes the repair and synthesis of coenzyme Q, and maintains the integrity of the mitochondria respiratory chain and the stability of mitochondrial function [57]. Mitofusin1/2 (Mfn1/2) is expressed on the outer mitochondrial member and plays a critical part in mitochondrial fusion [57, 58]. Optic atrophy 1 (Opa1) belongs to the dynamin superfamily and locates on the mitochondrial inner membranes. The Opa1-null cells present the form of fragmented mitochondria due to abortive fusion, although the outer membrane is successfully fused after mitochondrial fission [57].

It is believed that Nrf2 could increase proteasome activity, degrade Drp1, contribute to mitochondrial hyperfusion and thus exert a protective effect against cell death [59]. The results from one PC12 cell OGD experiment showed that OGD significantly induced apoptosis and inhibited PC12 cells activity, while administration of 3-n-butylphthalide significantly increased Nrf2, enhanced Mfn1/2 and Opa1, promoted mitochondrial fusion, improved the morphological and functional abnormalities of mitochondria, and finally protected PC12 cells [55]. In addition, the activation of Opa1 induced by Nrf2 not only promotes the fusion of the mitochondrial inner membrane but also restores the mitochondrial cristae, so as to enhance the ability to cope with stress [49].

To sum up, Nrf2 restores mitochondrial dysfunction after cerebral I/R in various ways. The results from an aforementioned *in vitro* OGD cell experiment using PC12 cells could be used, sum up, all the procedures mentioned above. It was found that 3-n-butylphthalide activated the expression of Nrf2, enhanced the level of mitochondrion fission and fusion-related proteins, restored the reduced mitochondrial potential difference, inhibited cell apoptosis, and finally improved the cell viability after OGD [55].

### 3.2 Nrf2 Inhibits Oxidative Stress

Oxidative stress refers to the phenomenon that the content of ROS exceeds the scavenging ability of the organism. The brain always faces a great risk of oxidative stress due to its high energy requirement, relatively weak antioxidant capacity and abundant lipid content [60]. After cerebral I/R, the intracellular concentrations of Cu^2+^/Fe^2+^ are elevated significantly, which transforms H_2_O_2_ into toxic ·OH. Besides, ischemia also reduces the activity of oxidoreductase, breaks the electron transfer chain, evokes the electron leakage from the mitochondrial inner membrane, further oxidizes the increased O_2_ during reperfusion into O_2_·^-^ and aggravates the oxidative injury. In addition, the significantly decreased blood volume after cerebral I/R can compensatively enhance the secretion of NO from vascular endothelial cells to dilate blood vessels and augment the blood supply to the brain. However, along with the development of the disease, NO eventually over-accumulates and oxidizes O_2_·^-^ into a more active and unstable form ·OH, further inducing severe oxidative stress [61].

**Fig. (2):**
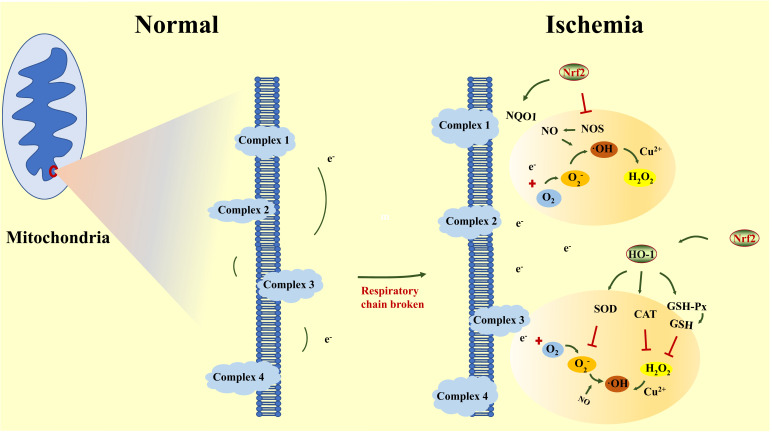
Nrf2 inhibits oxidative stress by inhibiting the formation of ROS and promoting the removal of ROS. **Abbreviations**: NQO1: NAD(P) H: Quinone oxidase 1; NOS: nitric oxide synthase; SOD: superoxide dismutase; CAT: catalase; GSH:glutathione; GSH-Px: glutathione peroxidase.

Oxidative stress is generally considered to be one of the most vital participants in the pathological change of cerebral I/R. On one hand, ROS induces lipid peroxidation of the cell membrane and generates malondialdehyde (MDA), thus aggravating the damage to BBB and other biological structures. On the other hand, ROS directly damages cyclophilin D (Cyp D), results in the opening of mPTP, enhances mitochondrial dysfunction and causes neuronal apoptosis [62]. Moreover, ROS also inhibits the ability of protease and ion channels, produces glutamate toxicity and calcium overload to exacerbate injury [63]. ·OH is considered one of the most active ROS and is believed to directly destroy the DNA structure of neurons, induce cross-linking breakage among proteins [64], facilitate the release of AIF and Cyt C from mitochondria and subsequently incur neuronal apoptosis [62]. Nrf2, which acts as an indispensable endogenous antioxidant participant, possesses significant anti-oxidative capacity. The antioxidant mechanism of Nrf2 can be divided into inhibiting the generation of ROS and promoting the removal of ROS (Fig. **2**).

#### 3.2.1 Inhibiting the Generation of ROS

##### 3.2.1.1 Inhibiting ROS Production

Emerging studies proved that astrocytes belong to a critical cell type that produce ROS during cerebral I/R injury. In an MCAO model study, 11-keto-β-boswellic acid (KBA) activated the Nrf2 pathway in a concentration-dependent manner, therefore significantly inhibiting the level of ROS derived from astrocytes and reducing the volume of cerebral infarction and alleviating the dysfunction of the brain. Meanwhile, in the cell experiment, knocking down Nrf2 or HO-1 of primary cultured astrocytes partly diminished KBA’s neuroprotective effect [65].

##### 3.2.1.2 Inhibiting the Secretion of NO

An *in vivo* transient cerebral I/R study demonstrated that andrographolide derivative CX-10 induced Nrf2 expression, down-regulated the expression of inducible nitric oxide synthase (iNOS), thus exhibited neuroprotective effects, including reduced cerebral infarction and improved neurological impairment [66].

#### 3.2.2 Promoting the Removal of ROS

##### 3.2.2.1 Enhancing the Expression of HO-1

An animal experiment proved that ficariside II boosted the expression of heme oxygenase 1 (HO-1) by activating Nrf2, and thus down-regulating the oxidative stress induced by cerebral I/R [67]. HO-1 can upgrade the production of CO to relieve neuropathic pain. Similarly, CO can also be used to induce or strengthen the activation of Nrf2, thus forming the positive loop between Nrf2 and HO-1 [68]. In addition, HO-1 also increased the activity of hemoglobin to catalyze biliverdin into bilirubin, which could enhance the expression of bilirubin’s downstream antioxidant proteins such as superoxide dismutase (SOD), catalase (CAT) and glutathione peroxidase (GSH-Px), to efficiently eliminate ROS [69]. The up-regulation of SOD facilitates the transformation of O_2_·^-^ into H_2_O_2_ and distinctly reduces O_2_·^-^ concentration after cerebral I/R [65, 70]. CAT up-regulation converts the increased H_2_O_2_ into O_2_, thus increasing O_2_ content while reducing H_2_O_2_ concentration. One research reported that Nrf2 activation increased the expression of CAT and SOD in rat brains, significantly enhancing their ability to scavenge ROS, and therefore effectively reducing MDA concentration [65]. GSH-Px facilitates GSH to combine with H_2_O_2_, and further reduces H_2_O_2_ to H_2_O [71]. Another study also clarified that curcumin-activated Nrf2, obviously increasing the concentration of GSH-Px, reducing the content of ROS, and further relieving oxidative stress after cerebral I/R [72].

##### 3.2.2.2 Enhancing the Expression of Thioredoxin and Inhibits Thioredoxin-Interacting Protein

Thioredoxins (Trxs) is a kind of system consisting of enzymes with disulfide reductase activity, including Trx, NAD(P)H and thioredoxin reductases (TrxR) [73]. TrxR can promote Trx to obtain two electrons from NAD(P)H, thus enhancing the capability of Trx to scavenge ROS [74]. However, thioredoxin-interacting protein (TXNIP) forms a heterodimer with Trx to prominently inhibit its antioxidant effect [75]. In addition, TXNIP activation also increases the expression of Nod-like receptor protein 3 (NLRP3) inflammasomes and contributes to the inflammatory response after cerebral I/R [76]. It is believed that the expression of Nrf2 positively correlates with TrxR. Increased Nrf2 expression enhanced TrxR activity and then decreased ROS level, while Knocking down Nrf2-induced TrxR reduction, increased cell apoptosis ratio, and inhibited cell proliferation [77]. A study claimed that Nrf2 activation significantly restrained the expression of TXNIP and alleviated oxidative stress and inflammatory infiltration, which could decrease the volume of cerebral infarct and improve the neurological function after cerebral I/R in a rat MCAO experiment [75].

### 3.3 Nrf2 Relieves Endoplasmic Reticulum Stress

The endoplasmic reticulum is not only the intracellular calcium reservoir that regulates the storage and release of intracellular Ca^2+^, but also the main place for synthesizing lipids and proteins [78]. After cerebral I/R, the deficiency of ATP synthesis and the accumulation of ROS pose severe threats to the endoplasmic reticulum, which leads to the corruption of endoplasmic reticulum structure and function, induces endoplasmic reticulum stress (ERS), including endoplasmic reticulum-associated degradation (ERAD) and unfolded protein effect (UPR), the latter decreases the level of misfolded proteins by repressing the synthesis of protein [79]. Glucose regulatory protein 78 (GPR78) possesses threonine kinase activity binds with three ER-related proteins: inositol-requiring enzyme 1 (IRE1), activating transcription factor 6 (ATF6) and protein kinase R–like ER kinase (PERK), and hinders their activity under physiological conditions. After cerebral I/R, a large number of misfolded proteins are synthesized in the endoplasmic reticulum, which activates GPR78 and dissociates GPR78 from IRE1, ATF6 and PERK. The phosphorylation of IRE1, ATF6 and PERK induced by oligomerization and autophosphorylation activates UPR, leading to the transient attenuation of global mRNA translation [80]. Physiologically, ERS acts as an endogenous protective way to inhibit oxidative stress and enhance the body’s adaptability to cope with injuries, but excessive ERS provokes calcium overload and over-inhibition of protein synthesis, finally limits self-recovery process [81]. Moreover, excessive ERS over-activates microglia and CD4^+^ lymphocytes, increases the number of M1 microglia and Th1 cells, injures myelin basic protein and neurofilament and significantly reduces the number of oligodendrocytes in corpus callosum [82].

It was reported that Nrf2 activation restrained the activity of GRP78 and halted its dissociation from ER-related proteins, further relieving excessive ERS after cerebral I/R [83]. Nrf2 can also prevent excessive ERS by inhibiting the activities of IRE1, ATF6 and PERK. The activation of the Nrf2 suppressed excessive ERS by inhibiting the activities of PERK, and thus down-regulated ATF6 expression in brain microvascular endothelial cells in a hypoxia and reoxygenation injury model [83]. Besides, the activation of Nrf2 also depressed IRE1, preventing the combination between PERK and eukaryotic initiation factor-2 (eIF2α), and subsequently repressing excessive ERS in a Wistar rat MCAO model [84].

### 3.4 Nrf2 Reduces Neuroinflammation

Neuroinflammation plays a central role in the pathogenesis of cerebral I/R injury [85]. Although neuroinflammation has beneficial effects on tissue repair in later stages, acute neuroinflammation, which is intimately linked to oxidative stress, inducing secondary neuronal damage, causing brain edema and aggravating cerebral I/R injuries [86, 87]. The primary features of neuroinflammation include peripheral immune cell infiltration, microglial activation, enhanced inflammatory cytokines, and local tissue damage.

Cerebral I/R induces vascular, cellular and molecular alterations, activates endothelial cells and pre-circulating leukocytes, up-regulates intercellular adhesion molecule-1 (ICAM-1), integrins and adhesion molecules including the E-selectins on the endothelial cell surface, P-selectins on platelet surface and L-selectins on leukocyte surface. Then, with the help of these molecular alteration, the leukocytes infiltrate into the brain parenchyma to secrete pro-inflammatory cytokines and cause inflammatory injuries [88]. Microglia are resident immune cells of the brain, and are activated by stimuli generated following cerebral I/R to retract their processes and present an amoeboid shape. The activated microglia include 2 phenotypes, including pro-inflammatory M1 and anti-inflammatory M2 phenotype. Microglia with M1 phenotype release inflammatory factors like tumor necrosis factor-a (TNF-a), interleukin-1β (IL-1β), interleukin-6 (IL-6) and ROS to exacerbate inflammatory injuries [86]. Human trails and animal experiments illustrate that the activation of inflammasomes may act as the initial part of the inflammatory reaction. Inflammasomes include NLRP1, NLRP3, NLRC4 and so on, among which NLRP3 is the most typical one. After cerebral I/R, NLRP3 spontaneously or secondarily sets off an inflammatory cascade reaction, up-regulating the expression of pro-inflammatory factors, causing intense inflammatory reactions and inducing leukocytes infiltration [89]. Nuclear factor kappa-B (NF-κB) is an ancient protein transcription factor and is widely expressed in neuronal cells, microglia, astrocytes and vascular endothelial cells. NF-κB combines with I-κB and keeps dormant under physiological conditions. Under stress conditions, NF-κB dissociates from p-I-κB and forms homodimers to enter the nucleus, which not only directly increases the expression of pro-inflammatory cytokines, but also activates NLRP3. The activation of NLRP3 and NF-κB plays a noteworthy initial role in the development of inflammatory cascade response, while activated microglia and infiltrated immune cells are the promoting elements during the aggravated inflammatory response after cerebral I/R [90-92].

**Fig. (3):**
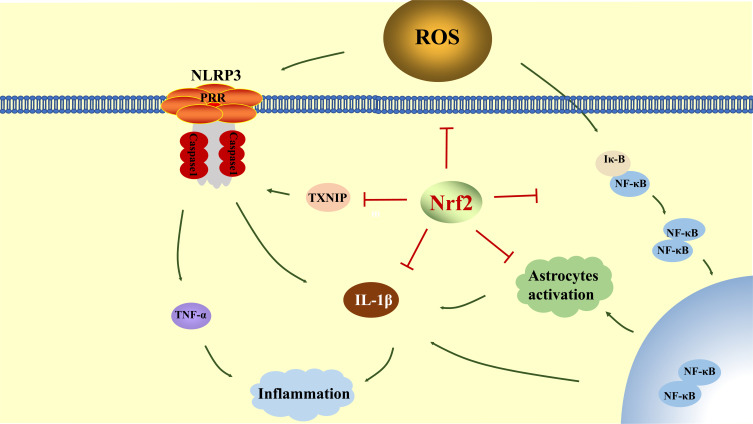
Nrf2 reduces inflammation by inhibiting NF-κβ, TNF-α and reducing the ROS. **Abbreviations**: NF-κβ: Nuclear factor-kappa β; TNF-α: tumor necrosis factor-α; TXNIP: thioredoxin-interacting protein; IL-1β: interleukin-1β.

A large number of studies have illustrated that Nrf2 exerts a significant inhibitory effect on inflammation after cerebral I/R: i) Nrf2 remarkably attenuates the activity of NLRP3. On one hand, Nrf2 effectively impairs the activity of NLRP3 by decreasing the production of ROS. On the other hand, Nrf2 also acts on Trx and TXNIP complex to distinctly attenuates the dissociation between TXNIP and Trx, thereby preventing the activation of NLRP3 by TXNIP [93, 94]. ii) Nrf2 precludes the phosphorylation of I-κB, and impairs the transcriptional activation of NF-κB, thereby limiting the inflammatory reaction induced by NF-κB [92]. iii) Nrf2 intensifies the activation of adenosine 5‘-monophosphate (AMP)-activated protein kinase (AMPK) to prevent the differentiation from anti-inflammatory M2 microglia to pro-inflammatory M1 microglia [95]. iv) Nrf2 reduces the induction of pro-inflammatory factors on adhesion molecules, selectin, vascular adhesion molecules (VCAMs) and ICAM-1 released by endothelial cells, leukocytes and platelets, further relieving the process of inflammatory reaction and subsequent damage on endothelial cells and the BBB [89] (Fig. **3**).

### 3.5 Nrf2 Repairs the Blood-Brain Barrier

BBB is an exceedingly specialized endothelial structure that is composed of microvascular endothelial cells, basement membrane, pericytes and astrocytes. BBB separates brain interstitial fluid (ISF) and cerebrospinal fluid (CSF) form peripheral circulation [96]. BBB that strictly regulates the movement of molecules and ions, and protects neurons and tissues from injury resulting from inflammatory factors or pathogens, is considered to be responsible for limiting peripheral toxins and maintaining the homeostasis of CNS [97]. After cerebral I/R, ROS and neuroinflammation induce the dysfunction of vascular endothelial cells, inspire the expression of selectin and ICAM-1, increase the activation of astrocytes and peripheral blood leukocytes, and promote inflammatory factors penetrating BBB and infiltrating into brain tissues [98]. Meanwhile, the increased ICAM-1 also damages tight junction proteins, increases the permeability of BBB, and facilitates the infiltration of neutrophils, thereby aggravating neuro-inflammation and cerebral edema [99, 100]. It was reported that the damage of BBB and the increase of BBB permeability might occur in hours after cerebral I/R [96].

Former studies proved that the activation of Nrf2 prominently restored BBB function after cerebral I/R. This protective effect may rely on promoting the expression of tight junction proteins (TJs) and blocking the activity of matrix metalloproteinase (MMPs).

#### 3.5.1 Promoting the Expression of Tight Junction Proteins

TJs bind with vascular endothelial cells, constitute the first shield of the BBB and limit the flow of water-soluble substances in peripheral circulation into the brain. In the most general case, TJs consist of a set of transmembrane (TM) proteins, including Zonula Occludens-1 (ZO-1), claudin and occludins, recruits additional structural proteins, signaling molecules, as well as the cytoskeleton to the cytoplasmic plaque, subsequently maintain the integrity of BBB [101].

An *in vivo* experiment claimed that the TJs, like ZO-1and occludin-5, were significantly decreased after MCAO/R, while the treatment with Nomilin up-regulated these expression of TJs to attenuate BBB disruption through the Nrf2 pathway, and therefore improved infarct area, brain edema and neurological deficits in a rat cerebral I/R experimental [102].

#### 3.5.2 Reducing the Expression of MMPs

MMPs are a highly congeneric family with a common core structure-zinc-dependent endoproteases and perform a variety of roles in tissue remodeling and protein degradation in the extracellular matrix (ECM). There are several MMPs subtypes, including MMP-1, -2, -3, -7, -8, -9, -12, -13, in which the MMP-9 subtype is dominant in endothelial cells [103]. Researchers examined the brain tissue, serum and cerebrospinal fluid and found that the significantly enhanced ROS promoted the expression of MMPs, increased the permeability of BBB and led to neuronal injuries.

An MCAO mouse experiment proved that the activity of MMPs was enhanced significantly in the acute phase of stroke (after the onset for 2-24 h), besides, this enhancement positively correlated with BBB permeability and cerebral infarct volume [104]. Another animal experiment showed that the activation of Nrf2-HO-1 pathway by osthole obviously decreased the cerebral concentration of MMP-9, amended BBB function, and enhanced the cognitive ability of experimental animals when compared with the global brain ischemia group [105].

### 3.6 Nrf2 Inhibits Excessive Neuronal Apoptosis

Apoptosis is a certain kind of programmed cell death process featuring by separation of the cell from the surrounding tissues, nuclear rupture, DNA fragment, chromatin and cytoplasm condensation, and phosphatidylserine exposure. Generally speaking, apoptosis can be induced by intracellular and extracellular toxins or stimulations [106] to eliminate the excessive proliferation or damaged cells and maintain the internal environment homeostasis [107].

It is found that the main injury of the ischemic penumbra neuron is reversible neuronal apoptosis, which quite differs from the irreversible neuronal necrosis in the ischemic core area after cerebral I/R. Therefore, the timely and effective inhibition of excessive apoptosis of penumbra neurons is an effective procedure to alleviate the neurological dysfunction of I/R patients [108]. In an OGD experiment with primary cultured cortical neurons, Acetyl-11-Keto-b-Boswellic acid (AKBA) treatment bolstered the binding of Nrf2 and ARE to significantly enhance its ability to clear away ROS, protected neurons form OGD-induced apoptosis. Meanwhile, in a rat MCAO experiment, AKBA increased the expression of Nrf2 in cortical neurons, reduced oxidative stress injury, shrank the size of cerebral infarction, and alleviated the deficits of neurological function. In contrast, the knocking down of HO-1 or Nrf2 reversed the alleviative effect of AKBA [109]. This study suggested that Nrf2 activation depressed excessive apoptosis of neurons after cerebral I/R.

**Fig. (4):**
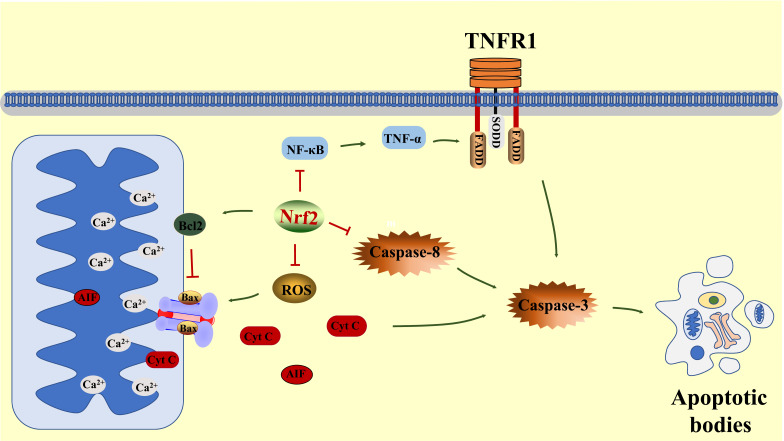
Nrf2 alleviates excessive neuronal apoptosis by inhibiting the endogenous apoptotic pathways and the exogenous apoptotic pathways. **Abbreviations**: Bcl2: B-cell lymphoma-2; AIF: apoptosis inducing factor; Cyt C: cytochrome c.

In detail, the inhibitory effect of Nrf2 on apoptosis can be divided into inhibiting exogenous apoptotic pathway and endogenous apoptotic pathways (Fig. **4**).

#### 3.6.1 Inhibiting Exogenous Apoptotic Pathways

Exogenous apoptotic pathway refers to the cascade reaction induced by the combination of activated death ligands and death receptors on the cell membrane surface under pathological conditions. After cerebral I/R, oxidative stress and inflammation activate macrophages to secret more TNF-α. The rising TNF-α combines with TNF-R1 receptors to recruit and activate initiator Caspase (Caspase-8). Then, the activated Caspase-8 recruits and activates executioner Caspase (Caspase-3) to combine with each other and cut down the carboxyl terminals, which motivates the formation of active Caspase-3, eventually incurs exogenous apoptosis reaction [110-112].

It is reported that Nrf2 activation is able to inhibit the activation of NF-κB pathway, decreasing the secretion of TNF-α and the trimerization of TNF-R1, restrain Caspase-8 activation, thus effectively hindering the activation of Caspase-3 [113, 114].

#### 3.6.2 Inhibiting Endogenous Apoptotic Pathways

mPTP is a complex protein composed of cyclophilin D (Cyp D), adenine nucleus transposase (ANT) and voltage dependent anion channel (VDAC). mPTP stands across the mitochondrial outer membrane and inner membrane confines the exchange of substance between the mitochondrial matrix and the surrounding cytoplasm. The increased ROS directly damages Cyp D, induces the opening of mPTP, releases Cyt C and AIF from mitochondrial matrix, causing or aggravating neuronal apoptosis after cerebral I/R [37, 38]. Besides, BH3, an apoptotic recognition factor protein of B-cell lymphoma-2 (Bcl2) family, is activated after cerebral I/R and then increases the expression of pro-apoptotic protein Bax to further promote the opening of mPTP [115].

The current studies showed that the inhibitory effect of Nrf2 on endogenous apoptotic pathways lies in the combination of Bcl2 to Bax. Activating Nrf2/HO-1 pathway enhanced Bcl2 to combine with Bax, prevented Bax from forming oligomer and creating pores on the mitochondrial outer membrane, reduced the release of AIF and Cyt C, inhibited the initiator Caspase (Caspase-9) activation, and further blocked executioner Caspase (Caspase-3). This blocked activation of Caspase-9 and Caspase-3 protected DNA from the damage caused by AIF and therefore attenuated cell apoptosis [113, 116, 117].

### 3.7 Nrf2 Repairs Neural Network Function

After cerebral I/R, insufficient ATP synthesis induces ion disorder, mitochondrial dysfunction, oxidative stress as well as inflammatory response, resulting in neuronal apoptosis and synaptic atrophy, subsequently leading to severe CNS dysfunction. Neural network repair includes angiogenesis, neurogenesis and synaptic formation, all of which are able to repair the neural network function and neural plasticity, and participate in improving the neurological functions in patients with stroke [118, 119].

#### 3.7.1 Promoting Angiogenesis

Angiogenesis refers to new collateral micro-vessels growing from existing blood vessels under the induction of vascular endothelial growth factor (VEGF), laminin and integrin. It often occurs within 4-7 days after cerebral ischemia, and promotes the recovery of cerebral blood flow and brain function, therefore reducing the recurrence rate of stroke patients [120]. Up to now, VEGF is the only discovered growth factor acting on endothelial cells to enhance angiogenesis [121]. One experiment using mouse brain microvascular endothelial cells illustrated that Nrf2 up-regulated the expression of VEGF, promoted the regeneration of new blood vessels and collateral circulation by activating phosphatidylinositol 3-kinase-protein kinase B (PI3K-Akt) pathway in the ischemic penumbra, thereby alleviating energy deficiency of the penumbra neuron in hypoxia condition. After knocking out Nrf2, the angiogenesis phenomenon was obviously impaired [122]. In addition, an MCAO rat model experiment proved that the *Cistanche deserticola* total glycosides activated Nrf2, increasing the contents of SOD and CAT, relieving oxidative stress, reinforcing the expression of CD31 and α-smooth muscle actin (α-SMA), facilitating angiogenesis in the ischemic penumbra, thereby eventually decreasing the infarct volume of cerebral I/R and improving neurological function [123].

#### 3.7.2 Promoting Neurogenesis

Neurogenesis could cement plasticity, restore neuronal signal transduction, promote myelination and the recovery of CNS function, and all these effects are primarily determined by the existence of adult NSCs. After being stimulated, NSCs proliferate and differentiate into neural progenitor cells, further migrating and differentiating into neurons, astrocytes, microglia and oligodendrocytes, to restore neural function [124, 125]. After cerebral I/R, due to the deficient nutrients and oxygen supply, the NSCs in the ischemic penumbra died in a short time [126], while the administration of tert-butylhydroquinone (tBHQ) was able to enhance the viability of NSCs by activating Nrf2 in an oxidative stress-induced cell death model [127]. Another study also proved that resveratrol not only attenuated NSCs injury, but also promoted NSCs proliferation through Nrf2 activation in an *in vitro* OGD/R experiment [128].

#### 3.7.3 Promoting Synaptic Formation

During cerebral ischemia, the loss of blood supply injuries the function of synapses. After reperfusion, the injury of astrocytes decreases the ability of astrocytes to transport glutamate, increases the content of glutamate, results in the glutamate toxicity, limits the transmission of synaptic inputs, and evokes or worsens the dysfunction of the synapse [129, 130]. Expression of synaptophysin, a presynaptic marker and postsynaptic density protein-95 (PSD-95), a major postsynaptic density protein, are believed to be synaptic markers [131], and cerebral I/R commonly leads to a marked decline in the expression of synaptophysin and PSD-95 [132].

An *in vivo* study discovered that the treatment with phenolic components of *gastrodia elata* activated Nrf2/ARE pathway in astrocytes to enhance the expression of brain-derived neurotrophic factor (BDNF), which could promote neuronal survival, differentiation, and synaptic plasticity after cerebral I/R [133]. The results from another rat cerebral I/R experiment showed that oleanolic acid could activate Nrf2 and further facilitate the expression of synaptophysin, synapsin and PSD-95, indicating that Nrf2 may help in promoting synaptic connection and neurogenesis [134].

In a word, Nrf2 exerts an ideal therapeutic role in improving the remolding of synaptic structures after cerebral I/R.

## 4 Nrf2 PARTICIPATES IN IMPROVING THE PROGNOSIS OF CEREBRAL I/R

Apart from being directly involved in the alleviation of cerebral I/R injury, Nrf2 may also be involved in improving the prognosis of cerebral I/R. Post-stroke cognitive impairment (PSCI) is a common complication of stroke and is characterized by a significant reduction in memory, attention and movement activity in stroke patients. The epidemiological survey suggests that stroke raises the incidence of cognitive impairment at least 5-8 times, and nearly 10% of patients with cognitive dysfunction possess a history of stroke, with the occurrence of repeated stroke up to 1/3 [135, 136]. Research reported that neuro-inflammation and oxidative stress caused by cerebral I/R were the main causes of cognitive dysfunction. A MCAO rat model experiment showed that after accepted rich environment therapies, the activated Nrf2-ARE pathway significantly increased SOD and CAT expression, reduced the content of MDA, and alleviated neuroinflammation caused by the over-activation of astrocytes, therefore restoring the brain function and improved recovery of cognitive ability at four weeks after stroke [137].

## CONCLUSION AND FUTURE PROSPECTS

Although Nrf2 is well known as a vital endogenous antioxidant factor, it also possesses the ability to repair mitochondrial dysfunction, alleviate ERS, inhibit the inflammatory response, reduce BBB permeability, alleviate neuronal apoptosis, enhance the remolding of neural networks and so on. Here, we prepare this review to explore the mechanisms underlying the pharmacological benefits of Nrf2 in relieving cerebral I/R, in order to provide the theoretical basis for exploring the role of Nrf2 activators in cerebral I/R.

Up to now, there are some Nrf2 activators applied in clinical applications. For example, a Nrf2 activator Bardoxolone-methyl was used for treating Alport’s syndrome diseases and diabetic kidney disease, and it was shown to exert ideal efficacy in the clinical trial. Another Nrf2 activator oltipraza is reported to prevent, intervene and mitigate colon and liver cancer, hepatic fibrosis and nonalcoholic fatty liver disease (NAFLD) [138]. Besides, there are numerous animal experiments illustrated the efficacy of ginsenoside, ginkgolide, tanshinone, ligustrazine, resveratrol and curcumin on cerebral I/R through activating Nrf2 pathway [139, 140], however, no clinical reports on their use in alleviating cerebral I/R injury was seen.

Therefore, there is still a long way to go in order to elaborate the role of Nrf2 activators in treating cerebral I/R. Here are some concerns: 1. Up to now, the evidence are still insufficient when considering whether Nrf2 could alleviate cerebral I/R injuries independently, or it exerts a synergistic effect with other pathways. This may affect the development of more specific targeted chemicals; 2. There is still a lack of comprehensive understanding of the specific structural information about Nrf2 and Keap1 [141], which may undermine the development of more potent Nrf2 activators; 3. The possible adverse effects of Nrf2 activators should also be considered. CDDO-Me is an effective Nrf2 activator, however, it induced heart complications in patients with end-stage renal disease in the clinical trials for treating diabetic nephropathy [142]. Besides, the long-term activation of Nrf2 may increase cancer risk, induce tumor metastasis as well as chemotherapy resistance, and deteriorate prognosis of cancer patients [143]. Thus, using other regimens to activate Nrf2 mildly might be a better choice. Utilizing hormestic dose response to induce endogenous Nrf2 expression by IPC may play a more effective role and prevent possible side effects of exogenous Nrf2 activator. 4. Based on the role of Nrf2 to alleviate oxidative stress and its protective effect in CNS, the use of Nrf2 agonists or treatments to induce endogenous Nrf2 may also play a therapeutic effect in some neurodegenerative diseases with oxidative stress as one of the pathological causes, such as Parkinson's disease or Alzheimer's disease [144, 145]. However, animal experiments and clinical trials are needed.

Although there are still many problems that need to be solved during the development of Nrf2 activators, previous studies have confirmed the significant value of Nrf2 activators. Therefore, the development of Nrf2 activators to alleviate cerebral I/R injury is worthy of further exploration.

## LIST OF ABBREVIATIONS

AIF: Apoptosis-inducing Factor
ANT: Adenine Nucleus Transposase
CAT: Catalase
CNS: Central Nerve System
CSF: Cerebrospinal Fluid
ERAD: Endoplasmic Reticulum-associated Degradation
ERS: Endoplasmic Reticulum Stress
GSH: Glutathione Family
iNOS: Inducible Nitric Oxide Synthase
IPC: Including Ischemic Preconditioning
ISF: Interstitial Fluid
mPTP: Mitochondrial Permeability Transition Pore
NSCs: Neural Stem Cells
ROS: Reactive Oxygen Species
SOD: Superoxide Dismutase
UPR: Unfolded Protein Effect
VCAMs: Vascular Adhesion Molecules
VDACs: Voltage-dependent Anionic Channels
